# The Fabrication of Biomimetic Chitosan Scaffolds by Using SBF Treatment with Different Crosslinking Agents

**DOI:** 10.3390/membranes1010003

**Published:** 2010-12-15

**Authors:** Chung-Tun Liao, Ming-Hua Ho

**Affiliations:** Department of Chemical Engineering, National Taiwan University of Science and Technology, No. 43, Sec. 4, Keelung Road, Da'an District, 10617 Taipei, Taiwan; E-Mail: M9806064@mail.ntust.edu.tw

**Keywords:** chitosan, biomimetic, SBF, osteoconduction, tissue engineering, crosslinking

## Abstract

In this study, a chitosan substrate was modified by simulated body fluid (SBF) treatment, in which the effect of the chosen crosslinking agent was investigated. Two crosslinking agents, glutaraldehyde (GA) and sodium tripolyphosphate (TPP), were used before the SBF process. By using TPP as the crosslinking agent, the Ca/P ratio and the degree of crystallinity were very close to the natural bone matrix. On the contrary, the substrate properties were very different from natural bone when the crosslinking agent GA was used. The results indicate that the produced substrates were more biomimetic when the TPP was applied. On the SBF-modified chitosan substrates with TPP crosslinking, the cultured osteoblastic cells expressed better proliferation, mitochondria activity and differentiation ability. The chitosan crosslinked using TPP was a good template in the SBF process, which resulted in a highly biomimetic layer. This biomimetic substrate possesses excellent biocompatibility and osteoconduction ability, promising high potential in the promotion of bone tissue engineering.

## 1. Introduction

Bone tissue engineering, a technique to create new bone tissue from cultured osteoblastic cells, has now been considered as a potential solution to the problem of bone loss. In bone tissue engineering, scaffolds or substrates, serving as transplanting vehicles for cultured cells and templates to guide tissue regeneration, play an important role in transforming the cultured cells into new tissues [1-3]. The development of scaffolds with suitable biocompatibility, cell/tissue affinity, mechanical properties, and handling characteristics is an attractive research topic. However, to make the scaffold more specific and effective to bone regeneration is one of the dominant factors in bone tissue engineering nowadays.

In the design of biomaterials, it is believed that nature is the best designer for organ or tissue development. Thus, one of the most promising approaches in scaffold preparation is to follow nature's design, which is to develop biomimetic substrates. For natural bone tissues, the most abundant organic substances in the extracellular matrix (ECM) are collagen and glycosaminoglycans (GAGs) [4]. Except for these organics, natural bone, having considerable contents of mineral components which give bone its hardness, is mainly a form of calcium phosphate. Matured bone contains about 65% mineralized matter, and the rest is collagen and matrix [4]. It has been proven that blending with collagen [5], GAGs [6] and calcium phosphate [7-9] during cell culture aids the attachment, proliferation or activity of osteoblastic cells. The osteoblastic phenotypes of cultured cells would be also improved by coating or grafting substances with collagen [5] and calcium phosphate [8,9]. It is reasonable to infer that the biomimetic modifications procured by the combination of these substances from the ECM can increase the osteoconduction effect of substances for bone tissue engineering.

Carbonateapatite, gelatin or the other bioactive molecules can be added into biomaterials by blending [7,9], coating [8], grafting [5] and so on; however, the immobilization of these bioactive molecules through simulated body fluid (SBF) provides many advantages over conventional methods [10-12]. It can easily create a surface layer of calcium phosphate on the biomaterials' surface without any complicated polymerization techniques. It can efficiently enhance the attachment and proliferation of some cells, especially osteoblastic cells [10,11]. In comparison to modification by dip coating or direct blending, SBF has been shown to enhance the substrates' biocompatibility more efficiently [10-12]. It is believed that the deposited layer formed by SBF possesses tiny structures that are very similar to the natural ECM in the human body. This technique has been applied to many kinds of surfaces of biomedical materials, including ceramics [11,13], metals [13,14] and polymers [13,15], and has also been used for the modification of scaffolds for bone tissue engineering [15].

To create a surface resembling human bone matrix, chitosan—whose molecular structure is similar to GAGs—was fabricated into substrates in this research. Firstly, chitosan has been identified to be helpful to the regeneration of bone tissues [15,17], due to the similarity between chitosan and GAGs, the main substance in the ECM of bones. Its properties of biocompatibility and long-term stability have also been revealed from previous studies [17,18] and are suited to our goals in this research. For the deposited surface layer in this research, the composition is designed according to natural bone tissues. Thus, gelatin, a protein product produced by partial hydrolysis of collagen, and calcium phosphate are deposited onto chitosan, a template for the guidance of the nano-structure in the biomimetic layer, by SBF for various periods.

Before the SBF process, chitosan must be crosslinked to increase its stability. GA (glutaraldehyde) is widely used for chitosan crosslinking because it is highly efficient and not expensive [15,16]. However, GA is not biocompatible enough to be used for biomedical applications and chitosan crosslinked with GA is usually too britle. In addition to the enhancement of stability and mechanical strength of chitosan, other template properties of chitosan substrates would be also controlled by the crosslinker, which was rarely noted in all the related studies. That is, the development of mineralized deposits would be restricted by the template molecule, chitosan. The properties of the deposited apatite layer may also influence the osteoconduction effects of chitosan substrates.

In this study, the chitosan substrate was prepared by freeze-drying and modified by the SBF bioreactor. All the details of the procedures were according to previous work [19] where the chitosan was used as a template for the formation of biomimetic nano-structures. To function as a template for ion-substituted carbonateapatite crystallization, the intermolecular distance must be controlled, thus sodium tripolyphosphate (TPP), which has never been used for chitosan substrates, was applied as a crosslinker in this research. The conventional crosslinker, GA, was also applied for comparison.

## 2. Materials and Methods

The chitosan substrate was prepared by casting/solvent evaporation technique [19], where acetic acid (0.5% w/v) was used as the solvent to prepare the chitosan solution of 1.5% w/v. Modification of the chitosan substrate with gelatin and TPP was formed by crosslinking for 2 h with TPP solutions. After the crosslinking process, the chitosan/gelatin substrates crosslinked by TPP were immersed in the simulated body fluid (SBF) for 14 days at 4 °C. For comparison, GA was used instead of TPP as a crosslinking agent, which forms colavent bonds, and all other steps were the same.

The SBF with modified formulation (1.5 × SBF) were prepared as described previously [1] by dissolving reagent grade NaCl, NaHCO_3_, KCl, K_2_HPO_4_·3H2O, MgCl_2_·6H_2_O, CaCl_2_, and Na_2_SO_4_ in deionized water. Previous researches have proved that the SBF solution, which has similar ionic composition to human body fluid, results in a deposited ion-substituted carbonateapatite layer on materials' surfaces, including glass, metal and polymer [15,16,20,21]. The pH value of SBF solution in this research was kept at 7. Finally, the chitosan/gelatin/TPP or GA ternary composite substrates were rinsed with deionized water at 4 °C to remove residual crosslinkers. After drying in air, the chitosan composite substrates were fabricated. After the ternary composite substrates were obtained, the modified substrates were immersed into SBF solution for different periods. After the chitosan substrates were fabricated, X-ray diffractometry (XRD), Scanning electron mictroscopy (SEM) and Energy dispersive spectrometer (EDS) nalyses were carried out to investigate substrates' properties.

The culture of osteoblast-like cells by using chitosan substrates was also performed. Cells were cultured in αMEM supplemented with 10% FBS, 1 mM sodium pyruvate and 100 U/mL penicillin-streptomycin-amphotericin, at 37 °C in 5% CO_2_. UMR-106 cells (rat osteosarcoma) were cultured in αMEM, supplemented with 10% FBS and 100 U/ml penicillin-streptomycin- amphotericin, at 37 °C in 5% CO_2_. The cultured cells were analyzed for the cell density, MTT assay and ALPase expression in this research.

After t incubation, the cultured cells were fixed with 0.2% glutaraldehyde and then with 2.5% glutaraldehyde at 4 °C. After that, cultured cells were dehydrated by using a series of alcohol solutions ranging in concentrations from 30% to 99%. Finally, critical point drying (CPD) was carried out before the SEM analysis.

## 3. Results and Discussion

**Figure 1:**
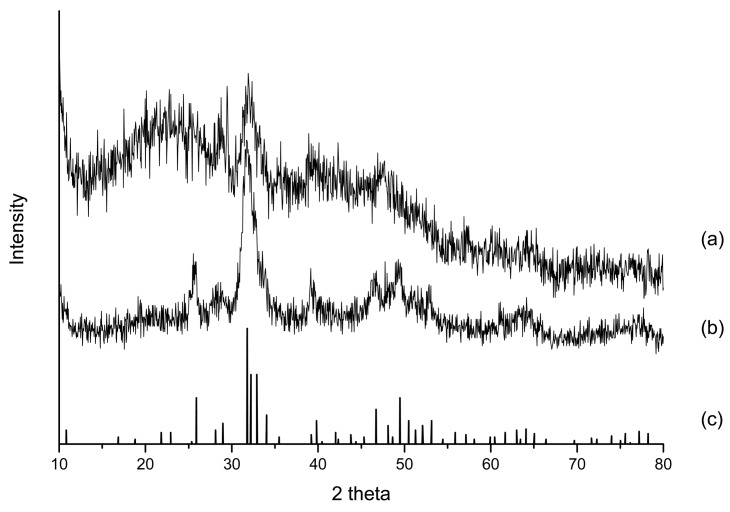
XRD spectra of (**a**) chitosan-gelatin composites crosslinked with TPP, (**b**) chitosan-gelatin composites crosslinked with GA, (**c**) hydroxyapatite from JCPDS # 09-0432. After the crosslinking, chitosan composites were treated with SBF for 14 days. The typical crystallization of ion-substituted carbonateapatite is indicated by the peak at 32 degree.

Figure 1 shows the XRD spectra of SBF-modified chitosan substrates rcrosslinked with GA (spectrum (a)) and TPP (spectrum (b)). Spectrum (c) in Figure 1 shows the XRD peaks of hydroxyapatite (JCPDS # 09-0432), the basic inorganic substance in the ECM of human bones. The spectrum (c) agrees with the result reported in the previous paper [22]. The results in Figure 1 indicate that no matter whether TPP or GA crosslinking was used, ion-substituted carbonateapatite clearly forms. The existense of ion-substituted carbonateapatite is confirmed from comparisons with spectrum (c). By Scherer's equation [6], the degree of crystallinity of SBF-modified chitosan scaffolds crosslinked by GA and TPP are 0.38 and 0.21. The crystallinity degree of TPP-crosslinked chitosan composites are very close to the crystallinity degree of natural hydroxyapatite *in vivo*, which is 0.26. On the other hand, by crosslinking with GA, the degree of crystallinity is much higher and not so close to the bone ECM. In contrast with GA-crosslinked substrates, the use of TPP is more advantageous for the formation of biomimetic ion-substituted carbonateapatite.

**Figure 2:**
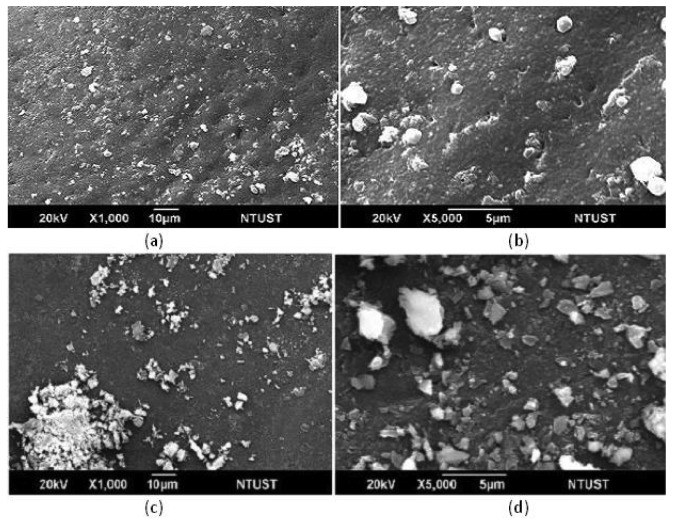
SEM images of the surfaces of chitosan-gelatin composites crosslinked with TPP ((**a**) and (**b**)) and GA ((**c**) and (**d**)). After the crosslinking, chitosan composites were treated with SBF for 14 days.

Figure 2 describes the surface morphology of chitosan composites, where a deposition layer of ion-substituted carbonateapatite forms after the SBF treatment. Comparing SBF-modified substrates crosslinked with TPP (Figure 2 (a) and (b)) and GA (Figure 2 (c) and (d)), we find that the former presents a surface which is more homogeneous with small crystals. On the contrary, GA crosslinking results in large, loosely-appearing deposits that aggregate in the surface deposition. After gentle washing, most of the deposits on the GA-crosslinked chitosan substrates come off the surface. These loosely-packed deposits easily depart from chitosan substrates in the cell cultures. On the contrary, the surface structure of TPP-crosslinked chitosan substrates remain intact with the deposition of ion-substituted carbonateapatite. The results reveal that the deposited layer of TPP-crosslinked chitosan would be more stable *in vitro* or *in vivo*.

In this research, the Ca/P ratio was determined by using EDS analysis, where the Ca/P ratios of chitosan substrates crosslinked with TPP and GA are 1.7 and 3.4, respectively. The Ca/P ratio of natural ECM in human bones is 1.67 [23,24] and very close to 1.7, which indicates that the properties of deposited ion-substituted carbonateapatite would be similar to the bone ECM. These results prove that the SBF-deposited surface layer is more biomimetic when TPP is used as the crosslinker. The previous paper also showed that osteoblasts express better attachment and proliferation on the substrates with a Ca/P ratio that is close to that of the natural ECM [23,24]. That is to say, TPP would be a better crosslinker to create a biomimetic surface on chitosan composites. This may be because TPP fixes chitosan molecular chains with a distance that is more similar to the natural size of inorganic crystals in human bones. Thus, chitosan crosslinked with TPP can serve as a template to develop a much higher biomimetic surface by SBF treatment, compared with chitosan crosslinked with GA.

**Figure 3:**
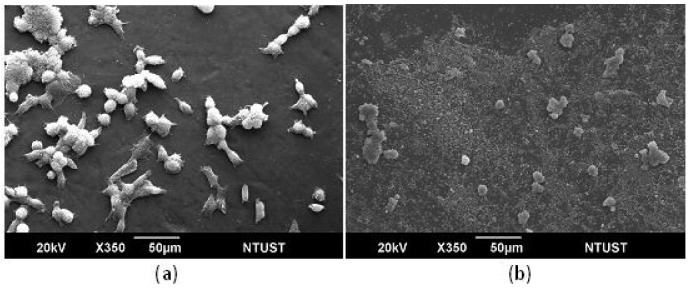
SEM images of osteoblast-like cells cultured on chitosan/gelatin/HA composites. **(a)** The deposited ion-substituted carbonateapatite layer is formed by SBF treatment after chitosan/gelatin is crosslinked with TPP; **(b)** the deposited ion-substituted carbonateapatite layer is formed by SBF treatment after chitosan/gelatin is crosslinked with GA. The period for the SBF treatment was 14 days. The cells were cultured for five days.

**Figure 4:**
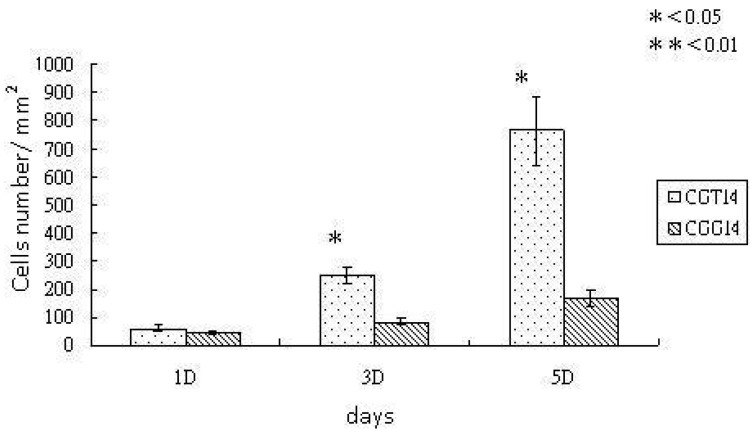
Cell density on chitosan/gelatin/ion-substituted carbonateapatite composite substrates crosslinked with TPP (CGT14) and GA (CGG14). After the crosslinking, chitosan composites were treated with SBF for 14 days. Significance (determined by t-test) is indicated as p < 0.05 (*) and p < 0.01 (**) based on the comparison between CGT14 and CGG 14.

To verify the biocompatibility and osteoconductivity of SBF-modified chitosan scaffolds, the *in vitro* experiments, cell proliferation and ALPase activity, were carried out. The proliferation of osteoblast-like cells, UMR-106, cultured with chitosan substrates were observed with SEM after an incubation time of five days. The SEM images are shown in Figure 3, and demostrate that the cell spreading is better on chitosan substrates crosslinked with TPP. The cells elongate well and had obvious lamapodia on the TPP-crosslinked chitosan substrates, while the cells on the GA-crosslinked substrates remained round without clear presence of attachment proteins. Previous studies have proven that the SBF-deposited layer on chitosan film crosslinked by GA can enhance cell proliferation significantly [15]. It is believed that the deposited ion - substituted carbonateapatite is osteoconductive. From Figure 4, the cell density observed on the TPP-crosslinked substrates is further higher than that observed on GA-crosslinked substrates. This is because TPP can make the SBF-deposited layer more biomemitic, which promotes the cell affinity of the chitosan substrates. The increase in biomimetic properties is proven by the measurements of crystallinity degree and Ca/P ratios.

**Figure 5:**
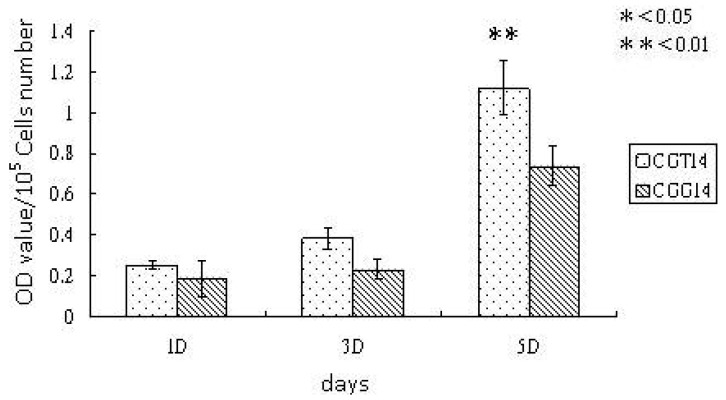
Cell viability on chitosan/gelatin/HA composite substrates crosslinked with TPP (CGT14) and GA (CGG14). Cell viability was measured by using the MTT assay. After the crosslinking, chitosan composites were treated with SBF for 14 days. The significance (determined by t-test) is indicated as p < 0.05 (*) and p < 0.01 (**) based on the comparison between CGT14 and CGG 14.

Cell viability, shown in Figure 5, was periodically measured by MTT analysis during cell culturing. Figure 5 indicates that cell viability was increased by the deposition of the layer formed by SBF treatment after the TPP-crosslinking. Kong *et al.*'s research has shown that SBF treatment on GA-crosslinked chitosan film promotes cellular activity [15]. Thus, the SBF-deposited layer on chitosan substrates with TPP-crosslinking more effectively promotes cell viability.

**Figure 6:**
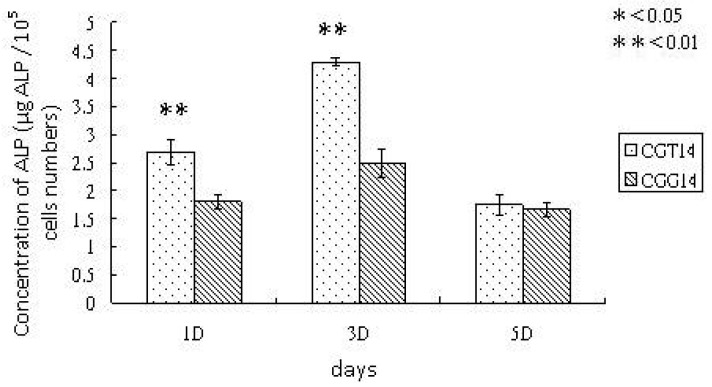
ALPase expression on chitosan/gelatin/ion-substituted carbonateapatite composite substrates crosslinked with TPP (CGT14) and GA (CGG14). After the crosslinking, chitosan composites were treated with SBF for 14 days. The significance (determined by t-test) is indicated as p < 0.05 (*) and p < 0.01 (**) based on the comparison between CGT14 and CGG 14.

ALPase activity increased during the early stages of osteoblastic differentiation and began returning to basal level in this culture [25]. The crosslinking effects of SBF-modified chitosan substrates on ALPase activity of osteoblastic cells is demonstrated in Figure 6. The continued expression and sufficient value of the early osteoblastic marker, ALPase activity, indicated that UMR cells attached to the modified chitosan substrates maintained the osteoblastic phenotype. Generally, ALPase expression first increased and then decreased over culturing time, as shown in Figure 6. These results suggest that the osteoblast-like cells could attach to chitosan substrates, and would be able to enter the further stages of bone differentiation. As demonstrated in Figure 6, the SBF modification on chitosan crosslinked with TPP enhanced the osteoinduction effects more than that crosslinked with GA; that is, the cells cultured on TPP-crosslinked and SBF-modified substrates expressed clearly higher ALPase activity. Results in previous studies [15] proved that the SBF-modifed chitosan crosslinked with GA is highly osteoconductive, revealed by enhanced ALPase activity and cellular mineralization. In comparison to the results in this research, it is demonstrated that applying TPP as the crosslinker instead of GA more effectively promotes osteoblastic differentiation. In summary, the *in vitro* experimental results of this study demonstrate that TPP is better than GA as a crosslinker for the SBF treatment on chitosan, as revealed by the higher cell proliferation rate, cell viability and ALPase expression on TPP-crosslinked chitosan/gelatin/ion-substituted carbonateapatite composite substrate. According to the XRD and EDS analyses, it is inferred that the chitosan substrate is controlled by the crosslinker, TPP, thus the deposited ion-substituted carbonateapatite crystals are more biomimetic, revealed by the crystallinity degree and Ca/P ratio which approximate the natural properties of human bones. The similarity of ion-substituted carbonateapatite depositition enhance substrates' cell affinity and osteoinduction effects more than the conventional SBF-modified chitosan substrates that are crosslinked with GA. That is to say, TPP could be applied to create a chitosan template for the formation of biomimetic ion-substituted carbonateapatite deposited layer in the SBF process.

Similar *in vitro* analysis have also been performed on the human gingival fibroblast (hGF), an osteoblastic cell line with multipotent properties. The results indicated that the cell proliferation, viability and ALPase expression are clearly enhanced by the ion-substituted carbonateapatite deposited layer on TPP-crosslinked chitosan substrates. The enhancement is greater than on GA-crosslinked chitosan substrates, corresponding to the results for UMR cells. However, these results are not presented in this study due to the limitation of paper length.

## 4. Conclusion

This research proposes a novel crosslinker, TPP, applied for the chitosan composite substrate before SBF treatment. The results prove that with TPP crosslinking, chitosan could serve as a template for the formation of ion-substituted carbonateapatite crystals in the SBF process. This deposited layer controlled with chitosan template is more biomimetic than those reported in the previous research [15, 16], revealed by the crystallinity degree and Ca/P ratio which are very close to those of natural human bones. The results of cell culture indicate that on the TPP-crosslinked chitosan substrate with ion-substituted carbonateapatite deposition, the spreading, proliferation, viability and ALPase expression of osteoblast-like cells, UMR-106, are all better than those on conventional SBF-modified chitosan crosslinked with GA. That is to say, the treatment developed in this research is improved over the conventional SBF processes which used GA as the crosslinking agent.
